# Postoperative Reduction in Postvoid Residual Urine Volume May Be Associated With Bladder Capacity Decrease Rather Than an Increase in Tidal Voided Urine Volume in Patients With Pelvic Organ Prolapse

**DOI:** 10.1111/jog.70358

**Published:** 2026-06-10

**Authors:** Kenji Kuroda, Hideaki Miyoshi, Koetsu Hamamoto, Kazuki Kawamura, Ayako Masunaga, Hiroaki Kobayashi, Keiichi Ito

**Affiliations:** ^1^ Department of Urology National Defense Medical College Tokorozawa Saitama Japan

**Keywords:** bladder capacity, laparoscopic sacrocolpopexy, pelvic organ prolapse, post‐void residual urine volume, tidal voided urine volume, transvaginal mesh surgery

## Abstract

**Aim:**

More than half of patients with pelvic organ prolapse (POP) complain of lower urinary tract symptoms (LUTS), especially voiding difficulty. Surgical approaches such as laparoscopic sacrocolpopexy (LSC) and transvaginal mesh surgery (TVM) often result in improved voiding function in POP cases. The present study primarily aimed to evaluate postoperative longitudinal changes in bladder‐related parameters after POP repair rather than determine superiority between surgical techniques.

**Methods:**

We retrospectively evaluated 216 patients with POP treated with LSC or TVM at our hospital. LSC was used for 86 patients, and TVM for 130 patients. Preoperative bladder capacity (BC), voided urine volume (VV), and post‐void residual urine volume (PVR) were recorded. The International Prostate Symptom Score (IPSS), quality of life (QOL) score, and 60‐min pad weight testing were also used to assess QOL change in LUTS.

**Results:**

BC significantly decreased 3, 6, and 12 months after LSC, and 6, 12 months after TVM. VV significantly declined 12 months after LSC but almost did not change compared with its preoperative value. PVR, IPSS, and QOL score significantly decreased 3–12 months postoperatively in both groups. Conversely, the rate of complications after surgery did not significantly differ between both groups. Comparison of preoperative condition and 1‐year postoperative outcomes showed no significant differences in PVR, 60‐min pad test, and IPSS plus QOL score.

**Conclusions:**

Our findings suggest that postoperative reduction in PVR may be associated with decreased BC at strong desire to void rather than increased tidal VV.

## 1 Introduction

Pelvic organ prolapse (POP) occurs when the pelvic floor support structures weaken, causing the pelvic organs to descend into the vaginal canal. Its cause is multifactorial, mostly due to the progressive weakening of the pelvic floor's connective tissues and muscles, which normally maintain the position of pelvic organs, such as the uterus, bladder, and rectum. According to population‐based studies, including those from routine gynecologic examinations, approximately 40% of women exhibit some degree of prolapse, ranging from stage 0 (no prolapse) at 24% to stage 3 (significant descent) at 3%; however, many cases remain asymptomatic and undetected without targeted evaluation [1].

Surgical intervention for POP aims to restore pelvic anatomy, alleviate symptoms, and improve quality of life (QOL), with options tailored to patient factors such as sexual activity, comorbidities, and prolapse stage. Laparoscopic sacrocolpopexy (LSC) and transvaginal mesh surgery (TVM) are two established surgical approaches for treating POP. LSC effectively restores anatomical structure and improves QOL, demonstrating significant improvements in pelvic floor distress and impact scores [2]. Owing to its favorable long‐term outcomes, LSC is often recommended for younger, highly sexually active patients [3]. In TVM, the operative time is generally shorter than that of LSC; this advantage is beneficial for patients who are at higher surgical risk, such as the elderly [3, 4]. TVM has been associated with a higher rate of mesh‐related complications, including mesh exposure and groin pain, although their prevalence is relatively low [2, 5]. Therefore, its use has been declining in the US and Europe because of its safety concerns [6]. Conversely, we have observed its effectiveness and safety; to date, we consider and apply TVM for POP repair as an important surgical option [4, 7].

More than 50% of women with POP report experiencing lower urinary tract symptoms (LUTS), which include urinary incontinence (UI), frequency, urgency, and voiding difficulties [8]. Especially, voiding symptoms, such as difficulty in urination and incomplete bladder emptying, are prevalent, affecting 73.8% of patients with POP [9]. POP can cause bladder outlet obstruction (BOO), leading to overactive bladder symptoms and detrusor overactivity. This obstruction may result from the anatomical displacement of pelvic organs, compressing the urethra [10, 11]. Surgical correction of prolapse often improves anatomical alignment, enhancing voiding function by reducing mechanical obstruction and improving bladder dynamics [12, 13, 14].

However, the mechanism underlying the recovery of voiding function to a near‐normal status remains unclear. In this study, we aimed to examine the course of improvement in preoperative voiding difficulty following surgical treatment, particularly LSC and TVM, in patients with POP, using several measurements, including bladder capacity (BC) and voided urine volume (VV).

## 2 Materials and Methods

### 2.1 Patients

We retrospectively enrolled 216 patients with POP treated with LSC (*n* = 86) or TVM (*n* = 130) at our hospital between December 2014 and November 2024. At our institution, LSC tended to be selected for younger and more sexually active patients because of its favorable anatomical durability, whereas TVM was often considered for elderly or higher‐risk patients because of its shorter operative time and lower surgical invasiveness. We examined them within the aforementioned period and followed them up for at least 1 year postoperatively at our institution. Indications for surgery included vaginal prolapse symptoms (pelvic organ prolapse staging system (POP stage) of ≥ 2 [15]), or POP‐induced hydronephrosis and/or hydroureter even in asymptomatic cases. All procedures performed in this study conformed to the tenets of the 2013 revision of the Declaration of Helsinki. The study protocol (Saitama, Japan; ID 4219) was accepted on August 21, 2020, by our institutional ethics committee, and all participants provided written informed consent. We included patients who underwent LSC or TVM for POP within the aforementioned period and excluded those who declined to participate.

The median follow‐up period after surgery was 12.6 months (interquartile range [IQR], 12.2–24.6 months). We collected data on surgical types, age, body mass index (BMI), POP stage, previous laparotomy, previous hysterectomy, blood loss, operative time, preoperative BC at strong desire to void, preoperative VV, preoperative post‐void residual urine volume (PVR), diabetes mellitus (DM) presence, and major intraoperative complications.

### 2.2 Surgical Procedure

One urologist performed LSC in accordance with the method reported previously [16]. Briefly, after port placement, the patient was positioned in a 15° Trendelenburg position to improve the visualization of the pelvic cavity by shifting the bowel cranially. The key steps were as follows:

1. Anterior vaginal wall dissection: The space between the anterior vaginal wall and bladder was dissected, and the anterior wall mesh—either Polyform (Boston Scientific Japan, Tokyo, Japan) or ORIHIME (Kono Seisakusho, Tokyo, Japan)—was secured.
2. Subtotal hysterectomy and bilateral adnexectomy: These procedures were performed if the uterus and adnexa were present.
3. Posterior vaginal wall dissection: The posterior vaginal wall was separated from the rectum, and the posterior wall mesh—either Polyform or ORIHIME—was secured.
4. Mesh retroperitonealization: The mesh was covered, and the pelvic peritoneum was partially closed.
5. Peritoneal closure: The peritoneum was completely closed after securing the mesh to the anterior longitudinal ligament at the level of the promontory.

Three urologists performed TVM according to the methods described previously [17, 18]. Briefly, after hydrodissection, the anterior vaginal wall was incised vertically, followed by full lateral dissection of the pubocervical fascia using a blunt technique to expose the sacrospinous ligaments. Then, the skin was incised by 4 cm laterally and 3 cm inferior to the anal center. A Shimada needle threaded with nylon monofilament sutures was subsequently advanced one or two finger breadths medial to the ischial spine to further penetrate the sacrospinous ligaments from the incision. After Polyform mesh or ORIHIME mesh arms were removed with nylon monofilament loops, an acceptable shape was created, spread, and secured under the bladder. The meshes were then cut to fit the stencil paper with two arms in advance. Finally, traction was employed over the externalized arms to ensure proper alignment, and the vaginal wound was closed using a 2‐0 Vicryl (Johnson and Johnson, Japan, Tokyo) continuous suture.

### 2.3 Assessment Methods for Preoperative and Postoperative Parameters

Prolapse recurrence was defined as the most dependent portion being at POP stage ≥ 2, which means that the most distal prolapse portion is ≥ −1 cm from the hymen plane, in accordance with Takazawa et al. [19] The occurrence of postoperative UI was defined by a daily life disturbance with UI. Mesh exposure was vaginally and/or visually examined using a vaginal scope.

Preoperative and postoperative BC, VV, and PVR were measured using uroflowmetry and TOTO Flowsky (TOTO Ltd. Research Institute, Chigasaki, Japan) when a patient felt a strong desire to void. Therefore, the measured BC in the present study reflects functional BC under physiologic filling conditions rather than formal cystometric BC obtained during filling cystometry.

The International Prostate Symptom Score (IPSS), QOL score, and 60‐min pad weight testing were used to assess QOL change in relation to LUTS until 1 year postoperatively.

### 2.4 Statistical Analysis

The Kruskal–Wallis test was performed to compare the differences in variable data between the LSC and TVM groups. Pearson's chi‐square test was used to determine the difference in the POP stage, previous laparotomy, previous hysterectomy, mesh product used, DM presence, intraoperative major complications, postoperative UI incidence, mesh exposure, and prolapse recurrence between the two surgical groups was determined using Pearson's chi‐square test. Statistical analysis was performed using JMP PRO (version 17; SAS Institute, Cary, NC). A *p* value < 0.05 was considered statistically significant.

## 3 Results

### 3.1 Patient Parameters

Table 1 shows the clinical and demographic characteristics of the patients enrolled in this study. Participants' median age was significantly lower in the LSC group than in the TVM group (74 vs. 75.7 years) (*p =* 0.0163). Likewise, the LSC group had a significantly lower BMI than the TVM group (22.6 vs. 24.8 kg/m^2^) (*p* < 0.0001). POP stage 4 was also significantly more prevalent in the LSC group than in the TVM group (17 vs. 14) (*p =* 0.0011). While previous laparotomy was more common in the TVM group than in the LSC group (3 vs. 12), the difference between these groups was not significant (*p =* 0.1041). Furthermore, previous hysterectomy was significantly more common in the TVM group than in the LSC group (37 vs. 9) (*p =* 0.0016). DM was also more prevalent in the TVM group than in the LSC group (23 vs. 10), but no significant difference was observed (*p =* 0.1146). Meanwhile, the median blood loss was significantly higher in the TVM group than in the LSC group (35.5 vs. 2 mL) (*p <* 0.0001). However, the median operating time was significantly shorter in the TVM group (1.1 vs. 3.1 h) (*p <* 0.0001).

**TABLE 1:** Clinical characteristics of the enrolled patients.

		LSC	TVM	*p*
		(*n* = 86)	(*n* = 130)	
Age (year), median (IQR)		74 (69–77)	75.7 ± 6.6	0.0163
BMI (kg/m^2^), mean ± SD		22.6 ± 2.4	24.8 (22.3–27.4)	< 0.0001
POP stage	Stage 2	6 (7.0%)	0 (0%)	0.0011
	Stage 3	63 (73.3%)	116 (89.2%)	
	Stage 4	17 (19.7%)	14 (10.8%)	
Previous laparotomy	Present	3 (3.5%)	12 (9.2%)	0.1041
	Absent	83 (96.5%)	18 (90.8%)	
Previous hysterectomy	Present	9 (10.5%)	37 (28.5%)	0.0016
	Absent	77 (89.5%)	93 (71.5%)	
DM	Present	10 (11.6%)	23 (17.7%)	0.1146
	Absent	76 (88.4%)	107 (82.3%)	
Blood loss (mL), median (IQR)		2 (0–9)	35.5 (19.5–66.8)	< 0.0001
Operating time (h), median (IQR)		3.1 (2.5–4.3)	1.1 (0.9–1.4)	< 0.0001
Preoperative bladder capacity at strong desire to void, mean ± SD		328.1 ± 148.5	291.7 (182.1–410.2)	0.4463
Preoperative voided volume, mean ± SD		260.5 ± 150.5	217.2 (117–330.3)	0.1341
Preoperative PVR, median (IQR)		0 (0–134.5)	66.5 (0–169.5)	0.0535
Mesh product used	Polyform	28 (32.6%)	38 (29.2%)	0.6033
	ORIHIME	58 (67.4%)	92 (70.8%)	
Intraoperative major complications	Present	10 (11.6%)	3 (2.3%)	0.0048
		AVW injury 7	Peritoneal injury 2	
		Bladder injury 3	Bladder injury 1	
	Absent	76 (88.4%)	127 (97.7%)	
LSC, laparoscopic sacrocolpopexy				
TVM, transvaginal mesh surgery				
IQR, interquartile range				
SD, standard deviation				
BMI, body mass index				
POP, pelvic organ prolapse				
PVR, post‐void residual urine volume				
DM, diabetes mellitus				
AVW, anterior vaginal wall				

Preoperative BC at strong desire to void was 328.1 mL (mean) in the LSC group and 291.7 mL (median) in the TVM group (*p =* 0.4463). Preoperative mean VV was also higher in the LSC group than in the TVM group (260.5 vs. 217.2 mL) (*p =* 0.1341). Conversely, preoperative median PVR was lower in the LSC group than in the TVM group (0 vs. 66.5 mL) (*p =* 0.0535). All three results showed no significant difference between the two groups.

Table 1 lists the major intraoperative complications. Bladder injuries, anterior vaginal wall injuries, and peritoneal injuries were repaired during surgery, and no serious postoperative complications were reported [20].

### 3.2 The Effects of LSC or TVM on Bladder Capacity and Postoperative Outcomes

**FIGURE 1:**
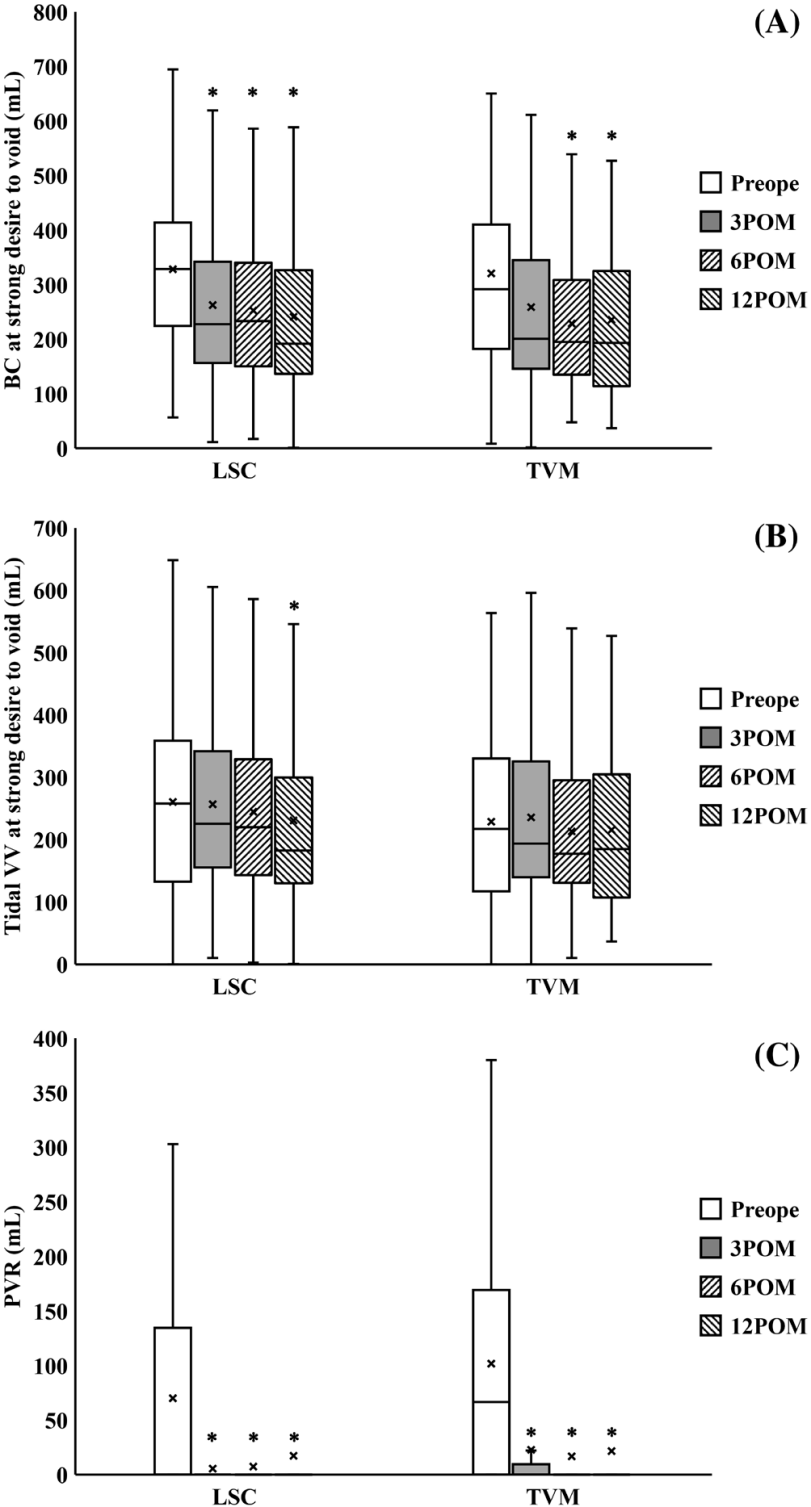
(A) Box plots reveal that BC at a strong desire to void significantly decreased 3–12 months after surgery (3 to 12 POM) in the LSC group and 6–12 months after surgery in the TVM group. * = significantly different from the preoperative period (Preope) (all *p <* 0.05). BC: Bladder capacity. (B) Box plots did not reveal any significant decrease in tidal VV at strong desire to void, except for 12 months after surgery (12 POM) in the LSC group. * = significantly different from the Preope (*p <* 0.05). VV: Voided urine volume. (C) Box plots reveal that PVR significantly decreased 3–12 months after surgery (3 to 12 POM) in both groups. * = significantly different from the Preope (all *p <* 0.0001). PVR: Post‐void residual urine volume.

BC significantly decreased 3, 6, and 12 months after LSC and 6 and 12 months after TVM (Figure 1A). VV significantly declined 12 months after LSC but almost did not change compared with its preoperative value and other postoperative variables in both groups (Figure 1B). Compared with other variables, PVR significantly decreased 3, 6, and 12 months after surgery in both groups (Figure 1C).

**FIGURE 2:**
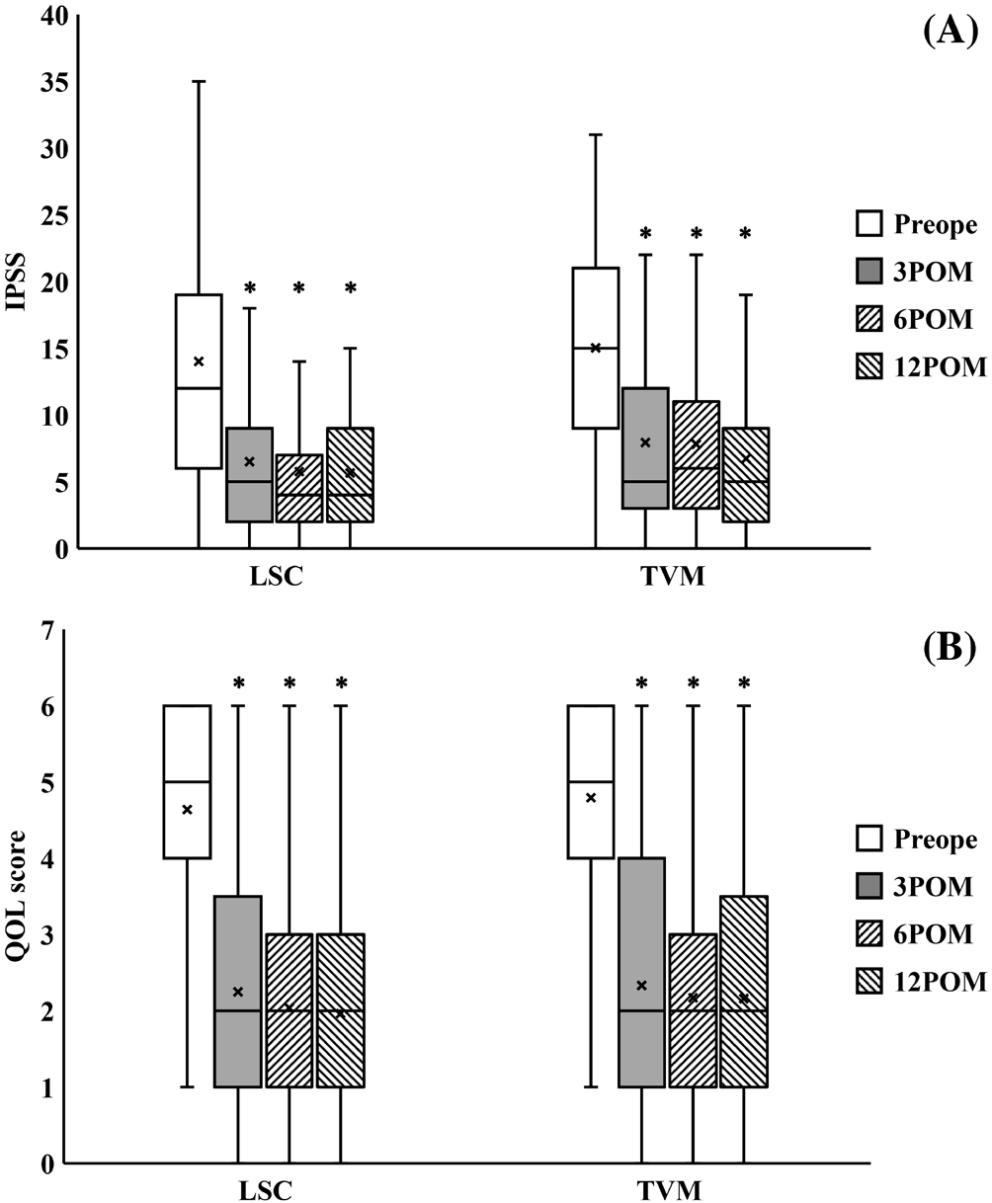
(A) Box plots reveal that IPSS significantly decreased 3–12 months after surgery (3 to 12 POM) in both groups. * = significantly different from the preoperative period (Preope) (all *p <* 0.0001). IPSS: The International Prostate Symptom Score. (B) Box plots reveal that the QOL score significantly decreased 3–12 months after surgery (3 to 12 POM) in both groups. * = significantly different from the Preope (all *p <* 0.0001). QOL: Quality of life.

IPSS and QOL scores significantly changed 3, 6, and 12 months after surgery compared with those before surgery in both groups (Figure 2A,B).

According to Pearson's chi‐square test, the postoperative rate of stress UI, mesh exposure, or prolapse recurrence did not significantly differ between the two groups (Table 2).

**TABLE 2:** Association between mesh surgical methods and the presence of incontinence, mesh exposure, prolapse recurrence after surgery.

		LSC (*n* = 86)	TVM (*n* = 130)	*p*
Incontinence	Present	9 (10.5%)	22 (16.9%)	0.8575
	Absent	77 (89.5%)	108 (83.1%)	
Mesh exposure	Present	1 (1.2%)	4 (3.1%)	0.2709
	Absent	85 (98.8%)	126 (96.9%)	
Prolapse recurrence	Present	5 (5.8%)	11 (8.5%)	0.4659
	Absent	81 (94.2%)	119 (91.5%)	
LSC, laparoscopic sacrocolpopexy				
TVM, transvaginal mesh surgery				

Comparison of preoperative condition and 1‐year postoperative outcomes by surgical technique showed no significant differences in PVR, 60‐min pad test, and IPSS plus QOL score (*p =* 0.9932, *p =* 0.183, *p =* 0.2478, *p =* 0.4149, respectively) (Table 3).

**TABLE 3:** Association between surgical methods and postoperative results at 12 months compared to preoperative conditions.

	LSC (*n* = 86)	TVM (*n* = 130)	*p*
PVR			
Worse	2 (2.3%)	3 (2.3%)	0.9932
Better or no change	84 (97.7%)	127 (97.7%)	
60‐min pad test	*n* = 84		
Worse	1 (1.2%)	3 (2.3%)	0.183
Better or no change	83 (98.8%)	127 (97.7%)	
IPSS			
Worse	0 (0%)	2 (1.5%)	0.2478
Better or no change	86 (100%)	128 (98.5%)	
QOL			
Worse	0 (0%)	1 (0.9%)	0.4149
Better or no change	86 (100%)	129 (99.2%)	
LSC, laparoscopic sacrocolpopexy			
TVM, transvaginal mesh surgery			
PVR, post‐void residual urine volume			
IPSS, international prostate symptom score			
QOL, quality of life score			

## 4 Discussion

In this study, age, BMI, POP stage, and hysterectomy history significantly differed between the LSC and TVM groups (Table 1). However, BC at a strong desire to void decreased 6 and 12 months after surgery in both groups, and VV did not change postoperatively, except for 12 months after surgery in the LSC group. In both groups, PVR significantly decreased 3, 6, and 12 months after surgery. These results may partly explain that BC reduction can lead to PVR reduction without increasing VV. Moreover, IPSS plus QOL score significantly improved postoperatively, with no significant differences in the rate of major complications and improvement in PVR, 60‐min pad weight, and IPSS plus QOL score between the two groups.

In this study, age, BMI, POP stage, and hysterectomy history significantly differed between the LSC and TVM groups (Table 1), reflecting differences in patient selection and surgical indications in routine clinical practice. Therefore, direct comparisons between surgical procedures should be interpreted cautiously because residual confounding and selection bias may have influenced postoperative outcomes. The main value of this study is the within‐group longitudinal observation of BC/VV/PVR dynamics.

Importantly, the BC evaluated in this study represents functional BC at strong desire to void rather than true cystometric BC measured during formal filling cystometry. Therefore, the postoperative decrease in BC observed in the present cohort may reflect normalization of bladder sensation or altered sensory thresholds after surgical correction of POP rather than a true reduction in anatomical bladder storage capacity. Patients with preoperative voiding dysfunction and elevated PVR may tolerate excessive bladder filling because of impaired bladder sensation or outlet obstruction. After surgery, recovery of more physiologic bladder sensation and improved emptying dynamics may lead patients to void at lower bladder volumes despite improved voiding efficiency. Accordingly, the observed reduction in functional BC should be interpreted cautiously and may partly reflect sensory recovery rather than deterioration of storage function.

Surgical intervention for POP can significantly impact BC and bladder function. Various surgical techniques, including LSC and TVM, have been studied for their effects on bladder dynamics. LSC can improve BC, with significant increases in the volume at first desire, strong desire, and overall BC, suggesting enhanced bladder's ability to store urine after surgery [21, 22]. TVM has been associated with a decrease in voiding difficulty and urine retention, suggesting an improvement in the bladder's ability to empty postoperatively. However, maximal BC notably decreased after surgery, indicating reduced ability of the bladder to store urine [23, 24]. In this study, BC at strong desire to void significantly decreased compared with other preoperative variables in both the LSC and TVM groups. Preoperatively, PVR tended to increase because of voiding difficulty, but after surgery, reduced voiding difficulty seemed to result in decreases in both BC and PVR.

Bladder compliance, a measure of the bladder's ability to stretch and accommodate urine, is an important factor when evaluating the surgical outcomes for POP. Robot‐assisted sacrocolpopexy (RSC) can increase bladder compliance, especially during the late filling phase [25]. This procedure minimizes the change in detrusor pressure; this feature is crucial for maintaining low‐pressure urine storage. Therefore, RSC may effectively restore bladder function by improving its compliance dynamics. LSC also minimizes detrusor pressure change during bladder filling, indicating improved bladder compliance and reduced bladder overactivity [25]. One study investigated the effects of TVM on bladder function and voiding workload and found that TVM diminished voiding resistance, lowered voiding pressure, elevated urine flow rate, and shortened the voiding time, but it did not affect the VV [26]. Their results indicated that bladder function recovered to the level that patients feel the desire to void with a normal urine storage volume postoperatively rather than a large volume, although bladder compliance increased as the voiding difficulty was alleviated. Bladder compliance increase and BC reduction at a strong desire to void appear to be consistent findings.

The change in PVR after surgery for POP may be influenced by several factors, including preoperative conditions, surgical techniques, and postoperative management. Patients with preoperative BOO are at a higher risk of persistent voiding dysfunction after surgery. In an earlier study, voiding dysfunction persisted in 9% of women with BOO 1 year after pelvic reconstructive surgery [27]. A preoperative maximal cystometric capacity of at least 500 mL and a PVR of at least 200 mL have been reported as significant predictors of postoperative voiding dysfunction [27]. Patients with preoperative voiding dysfunction, including a low urine flow rate (< 10 mL/s), are more likely to experience voiding dysfunction after surgery [14]. Our previous study also suggested that a PVR of at least 100 mL at 1 month postoperatively is a significant predictor of a PVR of at least 100 mL at 3 months postoperatively [13]. In this study, PVR significantly decreased postoperatively in both surgical techniques. Therefore, PVR may have decreased not because of increased tidal VV but because of decreased BC at a strong desire to void. This finding is considered a topic for future follow‐up.

In addition to the prolapse recurrence rate, the rates of mesh exposure or UI did not significantly differ between the LSC or TVM groups, consistent with some studies [28, 29]. One study revealed a very low rate of mesh erosion (2.6% for LSC and 3.1% for TVM), similar to our results (1.2% for LSC and 3.1% for TVM) [30]. In another study, UI developed in 24.1% of patients in the laparoscopic group and 35.8% in the vaginal surgery group preoperatively, and it persisted in 3.7% and 5.0%, respectively, after surgery [31]. In this study, UI persisted in 10.5% of patients in the LSC group and 16.9% in the TVM group; thus, UI was more frequently observed in this cohort than that reported above. Stress UI was the most frequent postoperative complication after either operation. In some studies, its prevalence ranged from 25% to 54.5% [32, 33, 34, 35]. In general, the previous UI rate is slightly higher than the present rate. The rates of prolapse recurrence were 5.8% in the LSC group and 8.5% in the TVM group in this study; these rates did not significantly differ from the earlier ones (5.0% for LSC and 8.6% for TVM) [36].

This study has some limitations. First, this retrospective analysis was initially conducted at a single institution, and the sample size was relatively small, limiting the generalizability of the findings. Second, significant baseline differences existed between the LSC and TVM groups, including age, BMI, POP stage, and hysterectomy history. Because adjustment analyses such as propensity score matching or multivariable regression were not performed, residual confounding may have affected intergroup comparisons. Third, formal filling cystometry was not performed in this retrospective study. Therefore, true cystometric BC, bladder compliance, detrusor pressure, and sensory thresholds could not be directly evaluated, limiting mechanistic interpretation of postoperative changes in BC. However, conducting multicenter studies with larger sample sizes and longer follow‐up periods incorporating flow cytometry could validate our findings and strengthen the evidence base. Moreover, future prospective studies using propensity score matching, multivariable regression analysis, or other adjustment methods may help minimize baseline imbalance and better clarify potential differences in postoperative bladder function outcomes between surgical procedures. Future investigations focusing on patients with high preoperative PVR or patients with suspected BOO, or correlations between objective PVR improvement and symptomatic improvement would also be required.

In conclusion, a decrease in PVR may be associated with a decrease in BC at a strong desire to void rather than an increase in tidal VV in both LSC‐ and TVM‐treated patients.

## Author Contributions

**Kenji Kuroda:** conceptualization, investigation, writing – original draft, methodology, visualization, project administration, data curation, software, formal analysis, writing – review and editing. **Keiichi Ito:** investigation, methodology, validation, project administration, resources, data curation, supervision, writing – review and editing. **Hideaki Miyoshi:** investigation, validation, methodology, project administration, data curation, formal analysis. **Ayako Masunaga:** investigation, methodology, validation, project administration, data curation. **Kazuki Kawamura:** investigation, methodology, validation, project administration, data curation. **Hiroaki Kobayashi:** investigation, validation, methodology, project administration, data curation, resources. **Koetsu Hamamoto:** investigation, methodology, validation, project administration, data curation.

## Disclosure

The authors have nothing to report.

## Ethics Statement

Ethical approval for this study protocol was granted by the Ethics Committee of the National Defense Medical College on August 21, 2020 (Saitama, Japan; ID: 4219).

## Consent

Written informed consent was obtained from all participants.

## Conflicts of Interest

The authors declare no conflicts of interest.

## Data Availability

The data sets produced and/or examined in this study were obtained from a single institution encompassing a restricted cohort of patients, thereby potentially facilitating the recognition of individual cases. Accordingly, in adherence to the Ethical Guidelines for Medical and Biological Research Involving Human Subjects in Japan, the data are rendered inaccessible to the public. The corresponding author may provide anonymized data upon a substantiated request and with approval from the Ethics Committee of the National Defense Medical College.
